# Discovery of Some Piperine-Based Phenylsulfonylhydrazone Derivatives as Potent Botanically Narcotic Agents

**DOI:** 10.1038/srep13077

**Published:** 2015-08-13

**Authors:** Huan Qu, Min Lv, Xiang Yu, Xihong Lian, Hui Xu

**Affiliations:** 1 Research Institute of Pesticidal Design & Synthesis, College of Sciences, Northwest A&F University, Yangling 712100, P. R. China; 2 College of Plant Protection, Northwest A&F University, Yangling 712100, P. R. China

## Abstract

By structural modification of piperine, some piperine-based phenylsulfonylhydrazone derivatives exhibited an unprecedented and potent narcotic activity against the oriental armyworm, *Mythimna separata* (Walker). The ND_50_ values of compounds 6c and 6e against the third-instar larvae of *M. separata*, which were more potent than those of wilfortrine and wilforgine, were 0.0074 *μ*mol (after 3.5 h), and 0.0075 *μ*mol (after 7 h) per larvae, respectively. By transmission electron microscope, it demonstrated that mitochondria were vacuolated and swollen in the ganglion cell of *M. separata* after treatment with 6c. More importantly, 6c selectively displayed the inhibition activity on acetylcholine esterase (AchE) of *M. separata*. This work paved the way for further studying the insecticidal mechanism of 6c as a new and promising botanical narcotic agent.

The oriental armyworm, *Mythimna separata* (Walker), is a major agricultural insect pest and hard to control in the world. Especially the sudden outbreaks of its larvae at very high densities could terribly result in complete crop loss^1^. Although chemical pesticides were widely introduced to control *M. separata*, the effects are not satisfactory. On the contrary, some serious problems such as the resistance and resurgence of *M. separata*, and environmental safety came into being^2^. Additionally, due to the advantages of botanical insecticides such as less or slower resistance development in pest species and lower environmental pollution^3^, the discovery of new insecticides directly or indirectly originated from plant secondary metabolites has recently received much research attention^4^,^5^,^6^.

Piperine (**1**, Fig. 1) was isolated as a simple alkaloid from *Piper nigrum* or an endophytic fungus of *P. nigrum*^7^, and displayed a broad spectrum biological activities., such as anti-inflammatory activity^8^, antimicrobial activity^9^, antitumor activity^10^, and insecticidal activity (contact toxicity, and growth inhibition)^11^,^12^, More recently, we have found piperine-based hydrazone derivatives displayed the potent delayed insecticidal activity^13^. To discover more potential natural-product-based insecticidal agents, herein we further prepared a series of piperine-based phenylsulfonylhydrazone derivatives as insecticidal agents. To our delight, interestingly, the piperine-based phenylsulfonylhydrazone derivatives exhibited an unprecedented and potential narcotic activity against *M. separata*.

## Methods

### Materials and Instruments

All chemical reagents were purchased and utilized without further purification. Solvents were used directly or treated with standard methods before use. Melting points (mp) were determined on a XT-4 digital melting point apparatus (Beijing Tech Instrument Co., Ltd., Beijing, China) and were uncorrected. Proton nuclear magnetic resonance spectra (^1^H NMR) and carbon nuclear magnetic resonance spectra (^13^C NMR) were recorded in DMSO-*d*_6_ on a Bruker Avance 400 or 500 MHz instrument using tetramethylsilane (TMS) as the internal standard. High-resolution mass spectra (HR-MS) were carried out with IonSpec 4.7 Tesla FTMS instrument. Compounds **2** (93% yield), **3** (91% yield), **4** (58% yield), and **5** (75% yield) were prepared in the same way as in our previous report^13^.

### General procedure for synthesis of piperine-based phenylsulfonylhydrazone derivatives (6a–f)

A mixture of compound **5** (80.8 mg, 0.4 mmol), phenylsulfonyl hydrazides (0.4 mmol) in ethanol (5 mL) was stirred at room temperature for 1–3 h. When the reaction was complete checked by TLC, the crude solid was collected by filtration, and washed with cooled ethanol (3 × 4 mL) and petroleum ether (3 × 4 mL) to afford compounds **6a–f** in 40–72% yields.

### Biological assay for narcotic activity (ND_50_, *μ*g/larvae) of compounds 6b–e^14^

The median narcotic dose (ND_50_, *μ*g/larvae) of compounds **6b–e** was evaluated by leaf disc method against the pre-third-instar larvae of *M. separata*. The acetone concentrations of **6b–e** were set as 2, 4, 8, 12, 16 and 20 mg/mL, respectively. For each concentration, 30 pre-third-instar larvae were used. Each larva was put into each well of cell culture plates and starved for 8 h. Then fresh wheat leaf discs (0.5 × 0.5 cm) were treated with 1 *μ*L of the above corresponding acetone solution. After drying, each piece of treated leaf discs was kept in each well of cell culture plates, which was placed in a conditioned room (25 ± 2 °C, 65–80% relative humidity (RH), 12 h/12 h (light/dark) photoperiod). Leaf discs treated with acetone alone were used as a blank control group. Avermectin was used as a positive control. During the process, the narcotic symptoms of the tested *M. separata* were observed, and the narcotic larvae were recorded at any time. Finally, the ND_50_ values of **6b–e** and avermectin were calculated against the pre-third-instar larvae of *M. separata*.

### Ultrastructural effect of compound 6c on the ganglion cell of *M. Separata* 14,15

First, the acetone concentration of compound **6c** was set as 50 mg/mL. Ten sixth-instar larvae of *M. separata* were used. Each larva was put into each well of cell culture plates and starved for 24 h. Fresh wheat leaf discs (0.5 × 0.5 cm) were treated with 1 *μ*L of the above corresponding acetone solution. After drying, each piece of treated leaf discs was kept in each well of cell culture plates, which was placed in a conditioned room (25 ± 2 °C, 65–80% relative humidity (RH), 12 h/12 h (light/dark) photoperiod). Leaf discs treated with acetone alone were used as a blank control group. After 8 h, the narcotic symptoms were observed. Second, the narcotic larvae were selected out. The ventral nerve cord was obtained from the dissected larvae. Third, the sample was pre-fixed with 4% glutaraldehyde solution (in 1% HCl buffer) at 4 °C for 0.5 h, and rinsed with 0.1 M phosphate buffer (pH 7.2) three times. Then the sample was treated with 1% osmium tetroxide fixative at 4 °C for 1.5 h, and rinsed with 0.1 M phosphate buffer (pH 7.2) three times. The sample was successively dehydrated by 30%, 50%, 75%, 95%, and 100% ethanol (each for 0.5 h), respectively. And it steeped twice in acetone (each for 0.5 h). Fourth, it was successively treated with Embed EPON-812 at different concentrations in acetone. It was then embedded with pure Embed EPON-812 in gelatin capsule, which was polymerized at 30 and 60 °C for 24 h, respectively. Finally, the 70 nm ultrathin sections of the sample were obtained by a LKB-NOVA slicing machine (Sweden), and the prepared sections were observed by the HT-7700 transmission electron microscope (Japan).

### Influence of compound 6c on the enzymes activities of *M. Separata* 16,17,18

The acetone concentration of compound **6c** was set as 15 mg/mL. Eighty fifth-instar larvae of *M. separata* were used. Each larva was put into each well of cell culture plates and starved for 12 h. Fresh wheat leaf discs (0.5 × 0.5 cm) were treated with 1 *μ*L of the above corresponding acetone solution. After drying, each piece of treated leaf discs was kept in each well of cell culture plates, which was placed in a conditioned room (25 ± 2 °C, 65–80% relative humidity (RH), 12 h/12 h (light/dark) photoperiod). Leaf discs treated with acetone alone were used as a blank control group. After 6 h, the narcotic symptoms were observed. Subsequently, the preparation of enzymes and assay for measuring enzymes activities described below: **a) Preparation of enzymes.** The heads of 50 narcotic fifth-instar larvae were dissected, weighed, homogenized (approx 10% weight/volume) with physiological saline in ice-cold condition using a teflon homogenizer, and centrifuged at 2500 r/min for 10 min at 4 °C. The supernatant was used as crude enzyme. Protein concentration of the supernatant was determined by the method of Coomassie brilliant blue G-250. The control group was treated in the same operation. **b) Assay for measuring Na**^**+**^**/K**^**+**^**-ATPase and Ca**^**2+**^**/Mg**^**2+**^**-ATPase activities.** The activity of Na^+^/K^+^-ATPase was assayed in the crude enzyme by determining the inorganic phosphate (Pi) liberated from the hydrolysis of the substrate adenosine triphosphate (ATP). The crude enzyme was diluted 10 times with physiological saline, and then measured the activity using Ultramicro Na^+^/K^+^-ATPase Diagnostic Kit. The operation method was according to the manufacturer’s instructions, and the absorbance was measured at 636 nm using ultraviolet spectrophotometer. The experiments were repeated three times. The activity of Ca^2+^/Mg^2+^-ATPase was also measured in an identical method using Ultramicro Ca^2+^/Mg^2+^-ATPase Diagnostic Kit. In this paper, ATPase activity unit was defined as capability of decomposing ATP of protein (mg^−1^) every 1 h into adenosine diphosphate (ADP) and Pi (*μ*mol^−1^). c) **Assay for measuring acetylcholine esterase (AchE) activities**. AChE activity was determined by measuring choline, the hydrolysate of acetylcholine, using AChE Diagnostic Kit. The operation method was according to the manufacturer’s instructions. In a 2.5 mL eppendorf tube, 0.04 mL crude enzyme, 0.5 mL substrate buffer, and 0.5 mL chromogenic agent were added. And then the mixture was incubated at 37 °C for 6 min in water bath. After incubation, 0.03 mL inhibitors, 0.1 mL transparent agent, and 0.05 mL stabilizers were added and mixed. The absorbance was measured at 412 nm using ultraviolet spectrophotometer. The experiments were repeated three times.

## Results and Discussion

**Figure 1:**
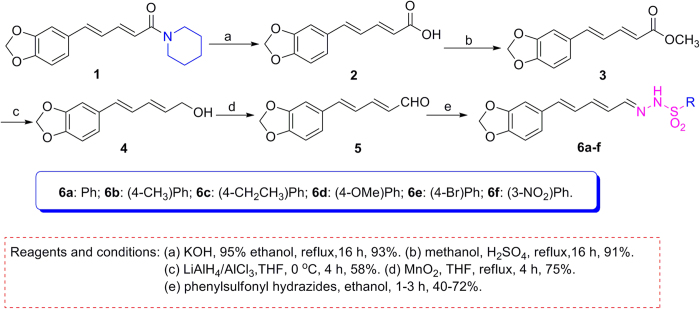
Synthetic route for preparation of piperine-based phenylsulfonylhydrazones (**6a–f**).

**Figure 2:**
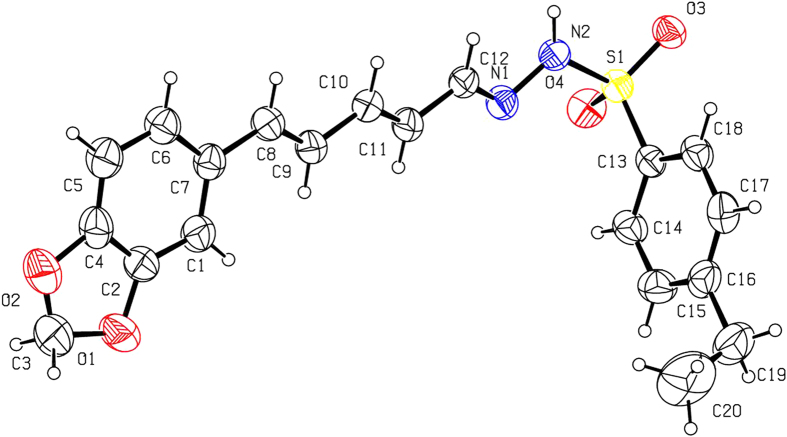
X-ray crystal structure of compound **6c**. Drawing by Hui Xu.

As shown in Fig. 1, basic hydrolysis of **1** gave piperic acid (**2**), which reacted with methanol to afford methyl piperate (**3**). Reduction of **3** in the presence of LiAlH_4_ and AlCl_3_ gave **4**, which was further oxidized by MnO_2_ to produce **5**. Finally, the target piperine-based phenylsulfonylhydrazone derivatives (**6a–f**) were smoothly obtained by reaction of **5** with different phenylsulfonyl hydrazides. All compounds were well characterized by ^1^H NMR, ^13^C NMR, HRMS and mp (see Supplementary data). To obtain the precise three-dimensional structural information of **6a–f**, the steric structure of **6c** was well determined by single-crystal X-ray diffraction (Fig. 2). The crystallographic data (excluding structure factors) for the structure of **6c** in this paper have been deposited with the Cambridge Crystallographic Data Centre as supplementary publication number CCDC 1002963. Copies of the data can be obtained, free of charge, on application to CCDC, 12 Union Road, Cambridge CB2 1EZ, UK [fax: +44 (0)1223 336033 or e-mail: deposit@ccdc.cam.ac.uk].

**Figure 3:**
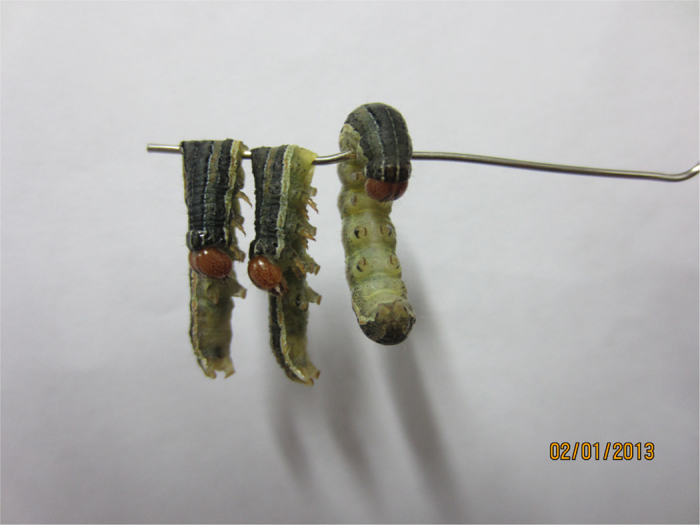
The narcotic symptoms of *M. separata* treated by compound **6c** (left and middle: narcotic larvae treated by **6c**; right: blank control group). Photograph by Huan Qu.

**Figure 4:**
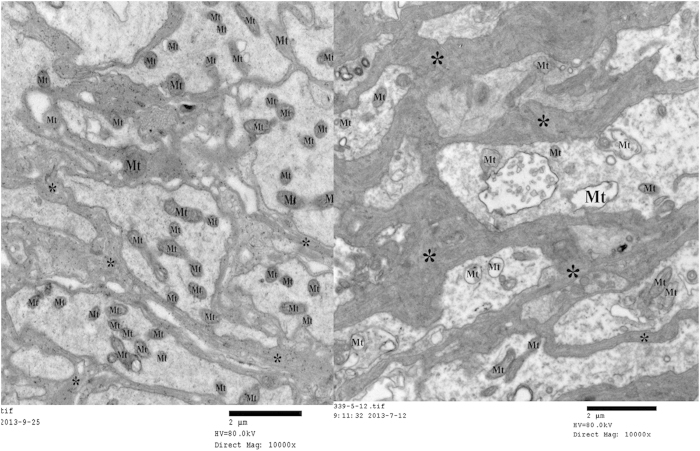
Ultrastructural effect of compound **6c** on the ganglion cell of sixth-instar larvae *of M. separata* (left: blank control group; right: treated group; Mt: mitochondria).

The narcotic activity of **6a–f** against *M. separata* was described in Table 1. Compounds **6c** and **6e** exhibited the strongest narcotic activity with the ND_50_ values of 0.0074, and 0.0075 *μ*mol/larvae, respectively. Compounds **6c** and **6e** were more potent than wilfortrine and wilforgine, two macrocyclic alkaloids isolated from *Tripterygium hypoglaucum* Hutch^19^. However, even if the larvae were treated by **1**, **6a** or **6f** at 20 mg/mL for 48 h, no narcotic symptoms of the tested *M. separata* were observed. According to the narcotic symptoms of the tested *M. separata* (Fig. 3), we proposed that **6c** and **6e** may act on the nervous tissue of *M. separata*. By transmission electron microscope, it clearly demonstrated that mitochondria were vacuolated and swollen in the ganglion cell of *M. separata* when treated by **6c** (Fig. 4).

**Figure 5:**
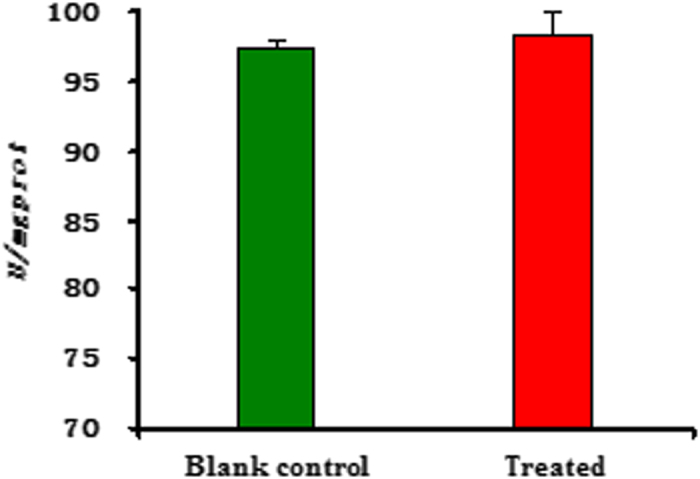
The change of activity (*U/mgprot*) of Na^+^/K^+^-ATPase on the head of the fifth-instar larvae of *M. separata* treated by compound **6c**.

**Figure 6:**
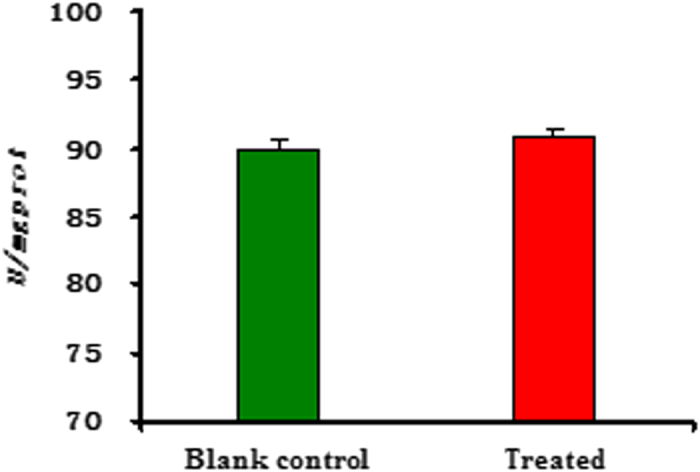
The change of activity (*U/mgprot*) of Ca^2+^/Mg^2+^-ATPase on the head of the fifth-instar larvae of *M. separata* treated by compound **6c**.

**Figure 7:**
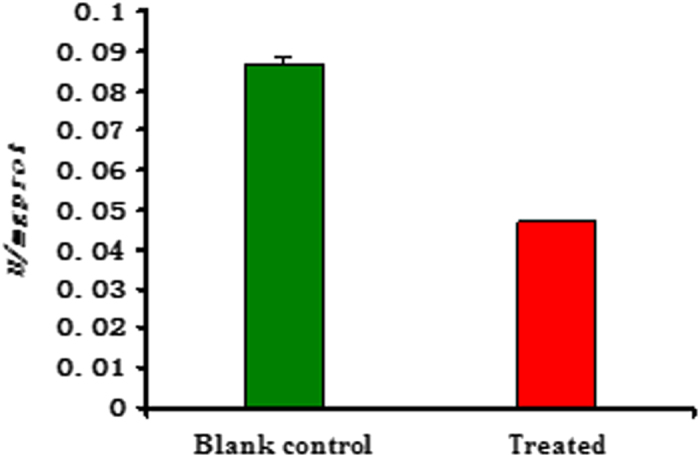
The change of activity (*U/mgprot*) of acetylcholine esterase (AchE) on the head of the fifth-instar larvae of *M. separata* treated by compound **6c**.

Additionally, we further investigated the activities of three main enzymes (such as Na^+^/K^+^-ATPase, Ca^2+^/Mg^2+^-ATPase, and acetylcholine esterase (AchE)) on the head of the fifth-instar larvae of *M. Separata* (see Supplementary data). As described in Figs 5 and 6, the changes of activity (*U/mgprot*) of Ca^2+^/Mg^2+^-ATPase and Na^+^/K^+^-ATPase of *M. separata* treated by **6c** were not very obvious; whereas there was an obvious change on AchE activity between the blank control group and the treated group (Fig. 7). It suggested that **6c** displayed the selective inhibition activity on AchE of *M. separata*. Therefore, it further demonstrated that **6c** acted on the nervous tissue of *M. separata*. To understand the reason of the narcotic activity of **6c** against *M. separata*, investigation of the mechanism on the nervous tissue of *M. separata* is currently underway in our laboratory.

## Conclusion

In summary, by simple structural modification of piperine, we have found some piperine-based phenylsulfonylhydrazone derivatives displaying the unprecedented and potential narcotic activity against the oriental armyworm, *M. Separata*. Among them, compounds **6c** and **6e** were more potent than wilfortrine and wilforgine. Compound **6c** displayed the selective inhibition activity on AchE of *M. separata*. It is noteworthy that for the chemical structures of **6c** and **6e** are very simple, total chemcial synthesis of **6c** and **6e** is also easier to realize when compared with wilfortrin, wilforgine and avermectins. Consequently, compounds **6c** and **6e** as the new and promising botanical narcotic agents, have the wide application prospect in the field of pesticidal chemistry.

## Additional Information

**How to cite this article**: Qu, H. *et al.* Discovery of Some Piperine-Based Phenylsulfonylhydrazone Derivatives as Potent Botanically Narcotic Agents. *Sci. Rep.* **5**, 13077; doi: 10.1038/srep13077 (2015).

## Supplementary Material

Supplementary Information

## Figures and Tables

**Table 1:** Narcotic Activity (ND_50_, *μ*mol/larvae) of Piperine-Based Phenylsulfonylhydrazones (**6a–f**) against the Third-Instar Larvae of *M. separata*.

**compound**	***t/*****h**	**Toxicity regression equation**	***r***	**ND**_**50**_
**6a**	/	**/**	**/**	**/**
**6b**	12	Y = 2.3821 + 2.7716x	0.9980	0.024
**6c**	3.5	Y = 3.9360 + 2.3441x	0.9730	0.0074
**6d**	12	Y = 2.2481 + 2.4539x	0.9740	0.0343
**6e**	7	Y = 3.3053 + 3.3160x	0.9727	0.0075
**6f**	/	/	/	/
**1**	/	/	/	/
wilfortrine^19^	8	/	/	0.0207
wilforgine^19^	8	/	/	0.0086
avermectin	0.5	Y = 4.4890 + 2.3660x	0.9958	0.0019
