# Supplementary material for: Discovery of Some Piperine-Based Phenylsulfonylhydrazone Derivatives as Potent Botanically Narcotic Agents
Source: Sci Rep. 2015 Aug 13;5:13077. doi: 10.1038/srep13077 (PMC4642516; doi:10.1038/srep13077)
Supplement: Supplementary Information [file srep13077-s1.doc]

**Supporting Information**

Discovery of Some Piperine-Based Phenylsulfonylhydrazone Derivatives as Potent Botanically Narcotic Agents

Huan Qu^1^, Min Lv^1,2^, Xiang Yu^1^, Xihong Lian^2^ & Hui Xu^1,2^

^1^Research Institute of Pesticidal Design & Synthesis, College of Sciences, Northwest A&F University, Yangling 712100, P. R. China.

^2^College of Plant Protection, Northwest A&F University, Yangling 712100, P. R. China.

*Corresponding author. Tel./fax: +86 29 87091952. *E-mail address:* [orgxuhui@nwsuaf.edu.cn](mailto:orgxuhui@nwsuaf.edu.cn) (H. Xu)

**1. Copies of ^1^H NMR and ^13^C NMR spectra**

**6a** ^1^H NMR

Date for **6a** ^13^C NMR

Date for **6b** ^1^H NMR

Date for **6b** ^13^C NMR

Date for **6c** ^1^H NMR

Date for **6c** ^13^C NMR

Date for **6d** ^1^H NMR

Date for **6d** ^13^C NMR

Date for **6e** ^1^H NMR

Date for **6e** ^13^C NMR

Date for **6f** ^1^H NMR

Date for **6f** ^13^C NMR

**2. All of the spectroscopic data of 6a-f.**

**6a**: Yield: 63%, pale yellow solid, m.p. 157-159 ^o^C; IR cm^-1^ (KBr): 3222, 3018, 2878, 1589, 1493, 1440, 1350, 1162, 1038, 993, 681; ^1^H NMR (500 MHz, DMSO-*d_6_*) *δ*: 11.35 (s, NH, 1H), 7.81 (d, *J* = 7.5 Hz, 2H), 7.60-7.68 (m, 4H), 7.16 (s, 1H), 6.88-6.94 (m, 3H), 6.67-6.73 (m, 2H), 6.23-6.28 (m, 1H), 6.03 (s, -OCH_2_O-, 2H); ^13^C NMR (125 MHz, DMSO-*d*_6_) *δ*: 149.20, 147.82, 147.45, 139.80, 138.98, 135.50, 132.88, 130.91, 129.12, 127.39, 126.98, 126.41, 122.18, 108.38, 105.29, 101.13. HRMS (ESI): Calcd for C_18_H_17_N_2_O_4_S ([M+H]^+^), 357.0904; Found, 357.0906.

**6b**: Yield: 63%, pale yellow solid, m.p. 172 ^o^C; IR cm^-1^ (KBr): 3208, 3020, 2898, 1599, 1494, 1441, 1354, 1163, 1042, 988, 671; ^1^H NMR (500 MHz, DMSO-*d_6_*) *δ*: 11.25 (s, NH, 1H), 7.69 (d, *J* = 8.0 Hz, 2H), 7.59 (d, *J* = 9.5 Hz, 1H), 7.39 (d, *J* = 7.5 Hz, 2H), 7.15 (s, 1H), 6.88-6.94 (m, 3H), 6.67-6.72 (m, 2H), 6.22-6.27 (m, 1H), 6.03 (s, -OCH_2_O-, 2H), 2.07 (s, -CH_3_, 3H); ^13^C NMR (125 MHz, DMSO-*d*_6_) *δ*: 149.00, 147.82, 147.44, 143.27, 139.66, 136.11, 135.43, 130.92, 129.54, 127.46, 127.04, 126.43, 122.16, 108.38, 105.29, 101.13, 20.90. HRMS (ESI): Calcd for C_19_H_19_N_2_O_4_S ([M+H]^+^), 371.1060; Found, 371.1059.

**6c**: Yield: 40%, yellow solid, m.p. 157-158 ^o^C; IR cm^-1^ (KBr): 3197, 3023, 2885, 1601, 1494, 1444, 1354, 1164, 1040, 990, 665; ^1^H NMR (400 MHz, DMSO-*d*_6_) *δ*: 11.29 (s, NH, 1H), 7.71 (d, *J* = 8.0 Hz, 2H), 7.60 (d, *J* = 9.6 Hz, 1H), 7.43 (d, *J* = 8.4 Hz, 2H), 7.16 (d, *J* = 1.6 Hz, 1H), 6.88-6.94 (m, 3H), 6.67-6.74 (m, 2H), 6.22-6.28 (m, 1H), 6.03 (s, -OCH_2_O-, 2H), 2.64 (q, *J* = 7.6 Hz, 2H), 1.16 (t, *J* = 7.6 Hz, 3H); ^13^C NMR (100 MHz, DMSO-*d*_6_) *δ*: 149.81, 149.58, 148.39, 148.01, 140.29, 136.90, 136.02, 131.48, 129.02, 128.03, 127.71, 127.00, 122.77, 108.96, 105.84, 101.71, 28.49, 15.53. HRMS (ESI): Calcd for C_20_H_21_N_2_O_4_S ([M+H]^+^), 385.1216; Found, 385.1224.

**6d**: Yield: 67%, yellow solid, m.p. 140-142 ^o^C; IR cm^-1^ (KBr): 3195, 3015, 2886, 1595, 1495, 1447, 1353, 1159, 1044, 990, 675; ^1^H NMR (500 MHz, DMSO-*d*_6_) *δ*: 11.17 (s, NH, 1H), 7.73 (d, *J* = 9.0 Hz, 2H), 7.59 (d, *J* = 10.0 Hz, 1H), 7.15 (s, 1H), 7.11 (d, *J* = 9.0 Hz, 2H), 6.88-6.94 (m, 3H), 6.67-6.72 (m, 2H), 6.22-6.27 (m, 1H), 6.03 (s, -OCH_2_O-, 2H), 3.83 (s, 3H); ^13^C NMR (125 MHz, DMSO-*d*_6_) *δ*: 162.48, 148.88, 147.83, 147.44, 139.57, 135.39, 130.93, 130.56, 129.23, 127.51, 126.45, 122.15, 114.28, 108.38, 105.30, 101.13, 55.57. HRMS (ESI): Calcd for C_19_H_19_N_2_O_5_S ([M+H]^+^), 387.1009; Found, 387.1019.

**6e**: Yield: 55%, yellow solid, m.p. 152-154 ^o^C; IR cm^-1^ (KBr): 3181, 3020, 2884, 1605, 1570, 1494, 1436, 1360, 1170, 991, 696; ^1^H NMR (500 MHz, DMSO-*d*_6_) *δ*: 11.42 (s, NH, 1H), 7.83 (d, *J* = 9.0 Hz, 2H), 7.73( d, *J* = 8.5 Hz, 2H), 7.62 (d, *J* = 9.5 Hz, 1H), 7.16 (d, *J* = 1.5 Hz, 1H), 6.89-6.95 (m, 3H), 6.68-6.75 (m, 2H), 6.23-6.28 (m, 1H), 6.03 (s, -OCH_2_O-, 2H); ^13^C NMR (125 MHz, DMSO-*d*_6_) *δ*: 149.68, 147.83, 147.48, 140.12, 138.17, 135.67, 132.26, 130.89, 129.02, 127.25, 126.85, 126.37, 122.22, 108.38, 105.32, 101.14. HRMS (ESI): Calcd for C_18_H_16_N_2_O_4_SBr ([M+H]^+^), 435.0008; Found, 435.0018.

**6f**: Yield: 72.9%, yellow solid, m.p. 174-176 ^o^C; IR cm^-1^ (KBr): 3169, 3025, 2892, 1604, 1532, 1495, 1440, 1359, 1173, 999, 670; ^1^H NMR (500 MHz, DMSO-*d*_6_) *δ*: 11.62 (s, NH, 1H), 8.50-8.53 (m, 2H), 8.23 (d, *J* = 7.5 Hz, 1H), 7.92 (t, *J* = 8.0 Hz, 1H), 7.65 (d, *J* = 9.5 Hz, 1H), 7.15 (s, 1H), 6.88-6.94 (m, 3H), 6.69-6.78 (m, 2H), 6.23-6.28 (m, 1H), 6.03 (s, -OCH_2_O-, 2H); ^13^C NMR (125 MHz, DMSO-*d*_6_) *δ*: 150.51, 147.84, 147.79, 147.53, 140.60, 140.37, 135.92, 133.07, 131.29, 130.85, 127.62, 127.07, 126.32, 122.29, 121.73, 108.39, 105.34, 101.16. HRMS (ESI): Calcd for C_18_H_16_N_3_O_6_S ([M+H]^+^), 402.0754; Found, 402.0765.

**3. The data of the activity changes (*U/mgprot*) of three main enzymes on the head of the sixth-instar larvae of *M. separata*** when treated by 6c.

| group | Na^+^,K^+^-ATPase | Ca^2+^,Mg^2+^-ATPase | AChE |
| --- | --- | --- | --- |
| Blank control | 97.41±0.63 | 89.97±0.79 | 0.08645±0.00195 |
| Treated | 98.23±1.86 | 90.84±0.60 | 0.04695±0.00041 |
